# *Trypanosoma Cruzi* Genome: Organization, Multi-Gene Families, Transcription, and Biological Implications

**DOI:** 10.3390/genes11101196

**Published:** 2020-10-14

**Authors:** Alfonso Herreros-Cabello, Francisco Callejas-Hernández, Núria Gironès, Manuel Fresno

**Affiliations:** 1 Centro de Biología Molecular Severo Ochoa, Consejo Superior de Investigaciones Científicas, Universidad Autónoma de Madrid, Cantoblanco, 28049 Madrid, Spain; alfonso.herreros@uam.es (A.H.-C.); bio.fcallejas@gmail.com (F.C.-H.); 2 Instituto Sanitario de Investigación Princesa, 28006 Madrid, Spain

**Keywords:** *Trypanosoma cruzi* strain, sequencing methods, genome plasticity, gene expression, trans-sialidases, mucins

## Abstract

Chagas disease caused by the parasite *Trypanosoma cruzi* affects millions of people. Although its first genome dates from 2005, its complexity hindered a complete assembly and annotation. However, the new sequencing methods have improved genome annotation of some strains elucidating the broad genetic diversity and complexity of this parasite. Here, we reviewed the genomic structure and regulation, the genetic diversity, and the analysis of the principal multi-gene families of the recent genomes for several strains. The telomeric and sub-telomeric regions are sites with high recombination events, the genome displays two different compartments, the core and the disruptive, and the genome plasticity seems to play a key role in the survival and the infection process. *Trypanosoma cruzi* (*T. cruzi*) genome is composed mainly of multi-gene families as the trans-sialidases, mucins, and mucin-associated surface proteins. Trans-sialidases are the most abundant genes in the genome and show an important role in the effectiveness of the infection and the parasite survival. Mucins and MASPs are also important glycosylated proteins of the surface of the parasite that play a major biological role in both insect and mammal-dwelling stages. Altogether, these studies confirm the complexity of *T. cruzi* genome revealing relevant concepts to better understand Chagas disease.

## 1. General Aspects of *T. cruzi* Biology

Trypanosomatidae family includes parasites of vertebrates, invertebrates, and plants. Due to their adaptation to different environmental conditions and high biological diversity, these protists produce a major impact on all biotic communities [1,2]. *Trypanosoma cruzi* (*T. cruzi*) is the parasite that causes the Chagas disease or American Trypanosomiasis, a chronic endemic illness of Central and South America, and a neglected tropical disease. Chagas disease is characterized by an acute phase with low mortality and symptomatology. Then, the patients can remain in an asymptomatic phase for life or, after many years without any sign of disease, develop a symptomatic chronic phase with cardiomyopathy, megavisceras, or both [3]. Moreover, these variations in the disease outcomes are related to the high genetic variability of the parasite [4,5,6,7].

*T. cruzi* presents a very complex life cycle that includes an invertebrate hematophagous triatomine vector and a broad range of mammalian hosts [8]. In both insect and mammalian hosts, four different major developmental stages were identified [9,10]. The non-infective epimastigotes are present in the midgut of triatomines where they differentiate into infective metacyclic trypomastigotes that after the infection of host cells are differentiated into the replicative amastigotes [11]. Finally, these amastigotes replicate by binary fission and lyse the cell differentiating to bloodstream trypomastigotes that can infect other cells of the host.

The mitochondrial DNA of *T. cruzi* is formed by a network of concatenated circular molecules of maxicircles and minicircles that is called the kinetoplast. This structure contains dozens of maxicircles (20–40 kb) and thousands of minicircles (0.5–10 kb) with varying sizes depending on species [12,13]. Maxicircles contain the characteristic mitochondrial genes of other eukaryotes and consist of two regions: the coding region and the divergent/variable region, very difficult to sequence due to its repetitive sequences [14]. Minicircles are exclusive to trypanosomatids and they are directly involved in U-insertion/deletion editing system as they encode guide RNAs (gRNAs) [15]. Moreover, it is suggested that both molecule populations are heterogeneous showing strain-specific variations [16,17].

*T. cruzi* reproduction is usually asexual by binary division, but there are evidences of natural hybridization, genetic exchange between strains and sexual reproduction [18,19,20,21]. Also, the population genetics of *T. cruzi* generated a significant interest, producing two opposing views. A clonal theory was proposed considering *T. cruzi* as the paradigm of the predominant clonal evolution (PCE) model of pathogens, displaying that this parasite shares many features with other parasitic protozoa, fungi and bacteria [22,23]. However, other researchers have demonstrated that *T. cruzi* could reproduce sexually by a mechanism consistent with classic meiosis, and have suggested that the PCE model in this parasite does not reflect the biological reality [21,24].

In mitosis the genome of *T. cruzi* does not condense to form chromosomes, preventing its visualization by conventional techniques [25,26]. Instead, parasite karyotype was determined by molecular biology techniques, such as pulsed-field gel electrophoresis (PFGE) in combination with Southern blot. These studies revealed a large molecular variability in size and number of chromosomes between strains and even among clones of the same strain [27,28]. The parasite is usually described as diploid, and the size of chromosomes varies from 0.45 Mb to 4 Mb and the number from 19 to 40. Experiments by flow cytometry have estimated the genome size between 80 and 150 Mb [29].

## 2. Classification of *T. cruzi* Strains

**Figure 1:**
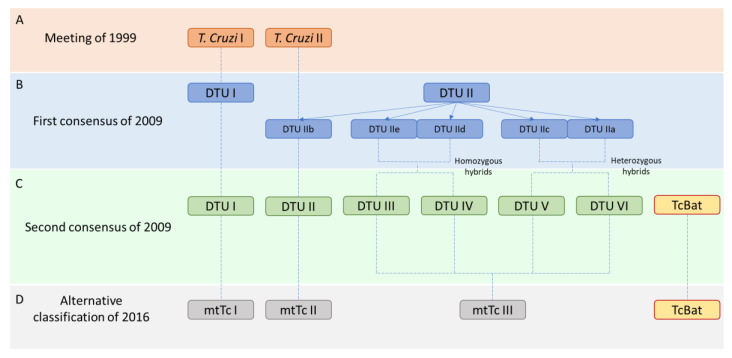
Different classifications of *Trypanosoma cruzi* since 1999. (**A**) Classification of the meeting of 1999. (**B**) First consensus classification of 2009. (**C**) Second consensus classification of 2009. (**D**) Alternative classification proposed in 2016.

There are many genetically different strains of *T. cruzi* [30,31]. Therefore, field investigators have looked for methods to classify these strains mostly according to their biological and genomic differences. The first classification was established in 1999 in a Satellite Meeting held at Fiocruz [32]. An expert committee reviewed the available data establishing two principal subgroups named *T. cruzi* I and *T. cruzi* II (Figure 1A). This classification was proposed considering biological and biochemical characteristics and molecular approaches such as the mini-exon studies and the 24Sα ribosomal DNA sequence.

Ten years later knowledge of the molecular diversity of the parasite increased and multilocus genotyping analyses revealed six distinct Discrete Typing Units (DTUs) [30], which in turn classified in two major subdivisions called DTU I and DTU II. DTUs are defined as “sets of stocks that are genetically more related to each other than to any other stock and that are identifiable by common genetic, molecular or immunological markers” [33]. Furthermore, based on phylogenetic information from multilocus enzyme electrophoresis (MLEE) and random amplified polymorphic DNA (RAPD) markers the DTU II was split into five DTUs (IIa-e) [34,35], and DTUs I and IIb correspond, respectively, to the *T. cruzi* I and *T. cruzi* II groups recommended by the original committee in 1999 (Figure 1B). This new classification considered that DTUs I and IIb were the ancestral strains, DTUs IId and IIe were the products of a minimum of two hybridization events [36,37,38], and DTUs IIa and IIc as ancestral hybrids.

However, a second revision that same year (2009) proposed a final classification in 6 DTUs [30]. DTUs I and II were the ancestral strains, DTUs III-IV those with at least one recombination event between DTUs I and II (homozygote hybrids), and DTUs V-VI were heterozygote hybrids of the DTUs II and III (Figure 1C). A new strain detected in bats was also included in the classification as TcBat [39] and with subsequent studies based on diverse molecular markers, it is considered to be the seventh DTU [40].

Finally, in 2016, Barnabé et al. [41] questioned the statistical validity of this classification. They performed a phylogenetic reconstruction by maximum likelihood trees based on the most common mitochondrial genes in databases. They proposed a new aggrupation considering the expression of three genes, two mitochondrial (*CytB* and *COII*) and one nuclear (*Gpi*). This new classification established three groups, the ancestral mtTcI and mtTcII, and the mtTcIII that grouped all the hybrid strains. They included the TcBat as an independent strain, although it was phylogenetically related to the mtTcI (Figure 1D).

## 3. The Genomes of *T. cruzi*: A New Update

The first version of a *T. cruzi* genome was published in 2005 [42] from the CL Brener strain. Interestingly, genomes for *Leishmania major* [43] and *Trypanosoma brucei* (*T. brucei*) [44] were simultaneously published in the same year.

The CL Brener strain was the most analyzed until then, with reproducible models in vitro, capable of producing an acute phase and being susceptible to Benznidazole [45]. In contrast to *Leishmania major* or *T. brucei* that had around 20–25% of repetitions in the genome, *T. cruzi* presented around 50%, making genome analysis and assembly more difficult [46]. Therefore, this first genome did not achieve the expected quality and remains incomplete, although it has been the principal reference for many researchers until today, despite the increasing availability of new and better genome sequences.

To date, there are several genomes of *T. cruzi* in the databases of the National Center for Biotechnology Information (NCBI) and TriTrypDB. This contributed to the study and understanding of the phenotypic, pathogenic, or complex variations among strains. Table 1 displays a summary of the recently available genomes in databases for the most studied strains. Some of these genomes were constructed from short-read sequencing methods (i.e., Illumina/Roche 454), such as Y [47], 231 [48], Sylvio X10/1 [49], G [50], or B7 strain of *T. cruzi marinkellei* [51]. Although these methods produce a high number of reads and have a low error rate, a relevant problem is the incapability to generate a complete chromosome reconstruction from short reads, causing very fragmented genomes in the case of complex genomes as trypanosomatids. This could lead to over-, under- or miss-representation of genes or complete chromosomic regions. In this regard, long-read sequencing methods (i.e., PacBio, Nanopore) could be a better choice for the trypanosomatids genomes [52], as the case of Bug2148 strain [53]. This technology allows the sequencing of long genetic fragments avoiding the complex and repetitive nature of the parasite. It could contribute to obtaining genomes with less redundant sequences and more completed, although the assembly size is still below the estimations made by DNA measurements (80–150 Mb) [29]. However, the error rate is bigger using long-read methods (and needs to be minimized by increasing the sequencing coverage) than in short-read methods. Therefore, in recent years, some laboratories chose the combination of both techniques to improve the assembly process, as the Berenice [54] or TCC and Dm28c [55] strains. In fact, the use of long-read sequencing methods generates contigs of more than 1 Mb, probably covering whole chromosomes. This allows the assembly of a genome in the smaller number of contigs, as happens with Berenice, Dm28c, TCC and Bug2148 strains (Table 1), obtaining the largest contig N50. Other researchers suggested that the copy number of conserved genes of *T. cruzi*, such as the monoglyceride lipase gene could be used as misassemble control [56].

Moreover, it was demonstrated that transcriptomic data may be useful to correct and re-annotate previous assembled genomes. Besides, in the case of Sylvio X10/1, RNAseq data was used to improve the previous genome annotation showing that 79.95% of the genome corresponds to the coding sequence, while the previous genomic analysis established only a 37.73% [57]. These results also suggested that the haploid genome for Sylvio X10/1 may be higher than previously reported (at least 51 Mb).

In the NCBI the reference genome is the hybrid CL Brener genome of 2005 [42,58] and presently many researchers rely on this information. CL Brener is a hybrid strain, where their homologous chromosomes presented different length and genetic content. Furthermore, this strain was separated in two haplotypes, named as Brener Esmeraldo-like and Brener Non-Esmeraldo-like, which genomes are also deposited in databases. Full length chromosome sequencing was performed with this hybrid strain, using a combined strategy based on bacterial artificial chromosome (BAC) ends sequencing and synteny maps with *T. brucei* [58], obtaining 41 virtual chromosomes (Table 1). Despite the continuous re-annotations of these genomes, they are far from being the best reference considering all the new and more completed genomes obtained with current techniques of long and short-read sequencing as Y [47], Bug2148 [53], Berenice [54] or Dm28c [55] strains. Therefore, we need to pose again which genome is appropriate as a reference for *T. cruzi* research and if the existence of just one genome reference is useful due to the high heterogeneity of the parasite. Moreover, and more importantly, some of the different DTUs of *T. cruzi* showed relevant differences in pathogenicity in mice [6]. This forces us to understand the differences at a genomic level and each strain would need a specific genomic analysis. Also, this high pathogenic, biological, and genetic diversity of the *T. cruzi* strains, even within DTUs, suggests that DTUs might not be a definitive form of classification, and it was hypothesized if *T. cruzi* could be a complex of species rather than a unique specie [59].

## 4. Genetic Diversity and Genome Structure of *T. cruzi*

### 4.1. Ploidy

Different studies confirmed the complexity of the *T. cruzi* genome, with different chromosome lengths between clones of the same strain, strains of distinct DTUs, or strains of the same DTU [26,28]. However, ploidy or chromosomal copy number variation (CCNV) analysis in this parasite could not be studied until the arrival of the Next Generation Sequencing (NGS) approaches.

Aneuploidy was studied in detail in *Leishmania*, whose “mosaic aneuploidies” are ploidy variations between isolates from the same strain and even between individual cells from the same population. These aneuploidies are related to drug resistance, gene expression regulation, or host adaptation [60,61,62]. Otherwise, in *T. brucei* a ploidy stability exists, including the subspecies *T. b. gambiense* and *T. b. rhodesiense* [63].

Regarding *T. cruzi*, the CCNV analysis depends on the quality of the assembled reference genome. Studies including strains of different DTUs revealed that as in *Leishmania*, the aneuploidy pattern varies among and within strains and DTUs [26]. However, the used reference genome was from CL Brener, which is not the most completed genome that we have in databases. Despite this limitation, it was concluded that the strains from DTU I seem to be more stable, while the strains from DTUs II and III present a high degree of aneuploidies as monosomies, trisomies, or tetrasomies [64].

These results suggest that the aneuploidies events could be used by *T. cruzi* to expand their genes and promote alterations in gene expression, something that may be critical for parasites that depend on post-transcriptional mechanisms to control gene expression. Although aneuploidies are mainly associated with debilitating phenotypes in many eukaryotes, they may be involved in species-specific adaptations during trypanosomatid evolution, affecting, for example, multi-gene families that are critical for the establishment of a productive infection in the mammalian hosts [65].

### 4.2. Genome Composition

Besides the different mechanisms to control gene expression such as polycistronic transcription, RNA editing, nuclear compartmentalization, or trans-splicing [66,67], *T. cruzi* presents genomic plasticity and an unusual gene organization among strains. Tandemly repeated sequences take up more than 50% of the *T. cruzi* genome and, although the parasite is considered a diploid organism, it presents variations in chromosome number and aneuploidy arrangements between strains and clones of the same strain [26,56,68].

The genome plasticity of *T. cruzi* is related to the genetic composition and a compartmentalization in two principal large regions of protein-coding genes was established. The first one is the core compartment, where we can find highly conserved genes with known function and genes without an assigned function typically annotated as hypothetical conserved genes that present synteny conservation with other species such as *Leishmania major* and *T. brucei*. The second one is the non-syntenic disruptive compartment, which is mainly composed by genes that evolve constantly, such as those that belong to surface multi-gene families (trans-sialidases, MASPs, or mucins). Both core and disruptive compartments show opposite G + C content and gene organization, with high differences in their regulatory sites [26,55].

*T. cruzi* genome is formed by three types of DNA. (1) Coding sequence of single-copy genes that are conserved between strains and species. (2) Coding sequence of multi-copy gene families, such as surface proteins or virulent factors. (3) Non-coding sequences and repetitive sequences, such as tandem repeats, retrotransposable elements and short repeat elements, which represent more than half of the genome affecting the methods of short-read sequencing above all as we explained before. Interestingly, around 50% of the genetic content of *T. cruzi* has unknown functions [47], which correlates with proteome studies of CL Brener, Dm28c, Y, and VFRA strains [69,70,71,72] in which around 40–50% of total proteins were of unknown function. This indicates how much we do not know yet about *T. cruzi* biology.

Regarding the single-copy genes, it was estimated that *T. cruzi* has more than 215 of these genes [54]. Although in the hybrid strains these genes might be underestimated according to previous results [47], due to the conservation of these genes and the apparition of new variations. Recent results in Y and Bug2148 strains confirmed this theory, with 183 and 400 detected single-copy genes, respectively [47]. The identification of these genes may help to understand the differential behaviors among strains as different pathogenicity, immune evasion, or life cycle.

### 4.3. Telomeric Regions

Telomeric and sub-telomeric regions in *T. cruzi* are sites of frequent DNA recombination that generate extensive genetic variations [73]. Therefore, they present a continuous evolutionary process. This concerns the relative abundance and organization of different genes, such as trans-sialidases, DGF-1 (dispersed gene family 1), RNA-helicases, RHS (retrotransposon hot spot genes), and N-acetyl-transferases [74,75]. In other protist parasites, as *T. brucei* or *Plasmodium falciparum*, sub-telomeric regions also present an important role in events of antigenic variation [76,77]. Trans-sialidase-like genes were located close to telomeric regions in *T. cruzi*, which generates new gene variations through non-homologous recombination. It was suggested that double-strand breaks produced in the sub-telomeric regions by retrotransposon nucleases are repaired by homologous recombination, but when the repair includes non-homologous chromatids there is a possibility to generate new gene variants [73]. This mechanism could contribute to the immune evasion of the parasite. Technically, it could also contribute to the collapsed assemblies of repetitive regions in sequencing. These sequences, the tandem repeats and/or other short repetitive genomic motifs, which correspond to telomeric and sub-telomeric regions, may produce an increment of fragmented genomes in *T. cruzi*, in comparison with other related species as *Leishmania* or *T. brucei*.

### 4.4. G + C Content

The %G + C is an indirect measure of the complexity of the genomes. Regarding the core and disruptive compartments of *T. cruzi*, they present a different content of G + C. While the core compartment has a 48% of G + C, the disruptive compartment has a 53%. In fact, it was hypothesized that genes with elevated recombination probability and constant evolution present high levels of guanines and cytosines [78,79]. We demonstrated in Y and Bug2148 strains that trans-sialidase-containing contigs (including pseudogenes) have a slightly higher %G + C content [47], suggesting that previous assemblies collapsed by repetitive sequences as those enriched in G + C [75,80]. These studies confirmed that variations in the %G + C were correlated with specific telomeric repeats described for *T. cruzi*, as the hexameric repeat TTAGGG and poly Ts structures [75,80]. Furthermore, in mammalian cells the %G + C content was correlated with mRNA expression, being the G + C-rich genes those with more efficient expression [81].

### 4.5. Replication Origin

Chromosomes of eukaryotic organisms are replicated from hundreds to thousands of DNA replication origins (ORIs), which are specified by the binding of the origin recognition complex (ORC). ORIs were mapped in *T. brucei* by marker frequency analysis sequencing (MFA-seq) coupled to ChIP analysis of the ORC [82]. These studies displayed that all mapped *T. brucei* ORIs are located at the boundaries of the transcription units. This was also detected in another specie as *Leishmania major*, where replication initiation sites are close to the genomic locations where the RNA pol II finishes, suggesting a strong correlation between the transcription kinetics and the replication initiation [83]. These studies also revealed more than 5000 potential sites of ORIs by SNS-seq techniques (Small nascent strand purification coupled with deep sequencing). However, another study detected by MFA-seq just one origin per chromosome in *Leishmania major* [84]. This happens because MFA-seq might detect mainly constitutive origins, while SNS-seq techniques may not reflect the frequency of origin activation, since these techniques might also identify flexible and/or dormant origins. Considering all those results, the complete replication of the genome in *T. brucei* and *Leishmania major* may require not merely constitutive ORIs that are fired in every cell cycle, but also further flexible and/or dormant ORIs, which cannot coincide with ORC binding and are fired stochastically [85].

Regarding *T. cruzi*, the ORIs of CL Brener strain were recently analyzed by MFA-seq [86], mapping 103 and 110 putative consensus ORIs in each haplotype of this hybrid strain. Moreover, the analysis displayed that some replication initiation sites map to the borders of the transcription units, as in *Leishmania major* and *T. brucei*. Interestingly, the majority of the putative predicted ORIs presented a great abundance within coding DNA sequences and showed a great G + C content enrichment (65% of average), while the genomic regions had a maximum of 54%. Also, another analysis with the same strain of *T. cruzi* by DNA combing, which can detect any replication initiation event (including constitutive, flexible and dormant origins, but without reference to genome location), displayed a median inter-origin distance of 1711 kb [87].

Considering the chromosomal location, while some ORIs of *T. cruzi* are located in non-transcribed regions as those seen in *T. brucei* and *Leishmania major*, many others are strategically localized at sub-telomeric regions (with a strong focus on DGF-1 genes), where they can produce genetic variability of multi-gene families [86]. The transcription orientation toward telomeres suggests that the abundance of putative ORIs in sub-telomeric regions produces head-on transcription-replication collisions since the replisomes go toward the centers of the chromosomes. These results suggest that collisions between DNA replication and transcription are recurrent in the *T. cruzi* genome and produce genetic variability, as suggested by the increase in SNP levels in the sub-telomeric regions and the DGF-1 genes containing putative ORIs [86].

## 5. Transcription of *T. cruzi*

Transcription in *T. cruzi* is polycistronic. Protein-coding genes are organized into non-overlapping clusters on the same DNA strand sometimes with unrelated predicted functions and separated by relatively short intergenic regions. Polycistronic transcripts are processed to produce mature mRNAs [88]. *T. cruzi* gene clusters can range from 30 to 500 kb separated by divergent or convergent strand-switch regions, or in a head-to-tail orientation whereby transcription terminates and then restarts from the same strand [57,89].

These strand-switch regions present a different nucleotide composition compared to the rest of the genome and a higher intrinsic curvature associated with transcriptional regulation [90]. In both *T. cruzi* and *T. brucei* canonical signals for RNA polymerase II promoters have not already been identified, except for the genes encoding the spliced leader (SL) [91]. In trypanosomatids, the transcription start sites and histone variants implicated in the transcription initiation process were described mainly at the divergent strand-switch regions [92,93]. Otherwise, the convergent strand-switch regions contain preferentially sites of transcription termination as well as RNA polymerase III transcribed tRNA genes [94].

**Figure 2:**
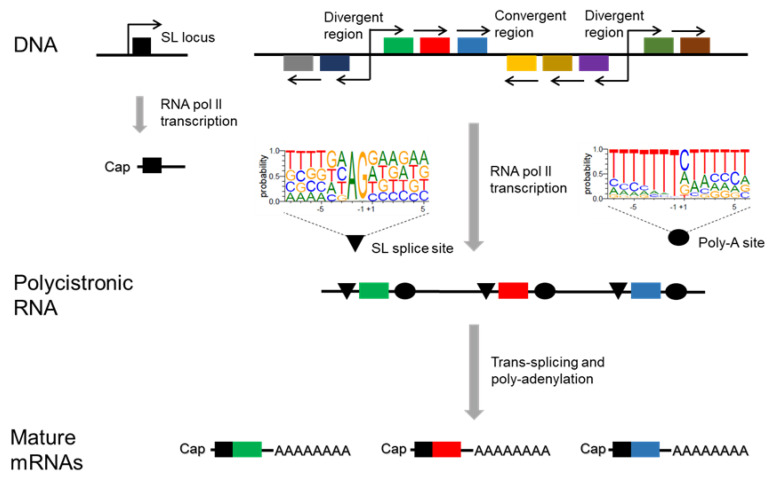
Transcription process of *T. cruzi*. RNA polymerase II produces polycistronic RNAs that are modified by trans-splicing and polyadenylation. The final mature mRNAs contain the Cap with the SL and the poly A tail. SL: spliced leader.

Up to hundreds of genes are transcribed at the same time by the RNA pol II in large Polycistronic Transcription Units (PTUs). The final mRNA maturation occurs by trans-splicing and polyadenylation processes (Figure 2). The trans-splicing is a special form of RNA processing by which two mRNAs encoded in different genome locations react to constitute a unique transcript [95]. In *T. cruzi* it consists of the insertion of a sequence of 39 nucleotides in the 5′ of each transcript, known as mini-exon or SL. This SL is transcribed from a tandem array as a precursor of around 140 nucleotides and is the target for the capping modification. The insertion of this Cap-SL gives stability to the mRNA and causes the excision of each mRNA of the PTU allowing the final polyadenylation [88,96].

The AG dinucleotide was described as the consensus sequence for the SL trans-splicing in *T. cruzi* [57], Leishmania major [97] and *T. brucei* [98]. However, small differences were detected between all of them in the nucleotide composition surrounding the AG dinucleotide, suggesting that different specific mechanisms are involved in the mRNA maturation among these species. For example, considering the first residue before the AG dinucleotide, the most probable in *T. cruzi* is an adenine, as in *T. brucei*, while in Leishmania major is a cytosine. Also, at position -4 a guanine is the most probable nucleotide in *T. cruzi* and Leishmania major, in contrast to *T. brucei* where a poly T tract starts and continues up to 50 nucleotides upstream. Interestingly, this pyrimidine enrichment is one of the principal differences between these trypanosomatids. In *T. cruzi* and *T. brucei* this C-T pattern is conserved just in the upstream 5′ region, while in Leishmania major represent about the 70% of the nucleotides upstream and downstream the AG dinucleotide. Besides, whereas the downstream region in *T. cruzi* is composed of purine nucleotides (A–G) up to 60%, in *T. brucei* A-T dinucleotides are the most frequent bases, indicating that *T. cruzi* and Leishmania major transcripts present a more proportional nucleotide composition than *T. brucei*.

The AAUAAA polyadenylation signal of eukaryotes is not present in trypanosomatids. Recent studies published by our group demonstrated that *T. cruzi* shows a single nucleotide that seems to be the most probable signal of polyadenylation start, being cytosine the most frequent nucleotide (45.3%) and thymine the less frequent (6.79%) [57]. This differs from other trypanosomatids species, as *Leishmania major* and *T. brucei* that presents a AA dinucleotide [97,98] as the most probable signal for polyadenylation. Furthermore, the surrounding genomic regions are also different. Whereas *T. cruzi* displays an abundant thymine composition in the upstream region, and a higher T-A composition in the downstream, *Leishmania major* shows a more variable sequence composition in both upstream and downstream regions, and *T. brucei* a uniform pattern in both extremes composed by T-A nucleotides. These results suggest that the mRNA maturation processes in *T. cruzi* may differ notably from *Leishmania major* and *T. brucei*.

Genes in trypanosomatids do not have promoter regions to regulate gene expression and their regulation is mainly at the post-transcriptional level, with a key role of the 3′ UTR regions. The principal mechanisms of regulation are the stability or instability of the transcripts, gene duplication, histone regulation, and translation efficiency [96,99]. Therefore, despite the genes of the same polycistron are transcribed in an equal proportion, differences in their expression were detected in distinct life cycle stages or growth conditions [97,98,100]. This could explain the selection of highly repetitive sequences in the parasite through evolution [46,101,102] by the aggregation of tandem repeats, retrotransposons, and repetitive short sequences in chromatin remodeling [103].

However, the concrete mechanisms involved in the regulation of the gene expression in *T. cruzi* are still unknown and were not further studied as in other species, such as *Leishmania* or *T. brucei* [104,105]. In this last specie, for example, it was demonstrated that in heat-shock conditions the genes close to the transcription initiation sites are down-regulated, while genes in a distal position increase their expression [106].

## 6. Principal Multi-Gene Families of *T. cruzi*

*T. cruzi* possesses several multi-gene families, some with hundreds of members, which contribute to the repetitive nature of the parasite’s genome, such as the retrotransposons or the tandem repeats. Most of these multi-gene families code for surface proteins, which play different key roles in the *T. cruzi* life cycle, from the establishment of an effective host-cell interaction and invasion until the protection against the host immune system. Furthermore, these multi-gene families present a huge expansion and constant evolution that produces a great diversity among strains [107].

**Figure 3:**
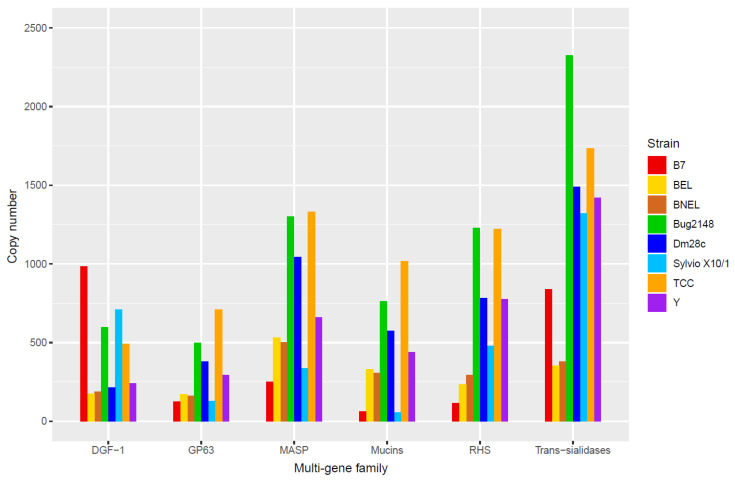
Genome copy number of the most abundant multi-gene families of *T. cruzi* and the B7 strain of *T. cruzi marinkellei*. BNEL: CL Brener Non-Esmeraldo-like; BEL: CL Brener Esmeraldo-like; DGF-1: Dispersed Gene Family 1; GP63: Glycoprotein 63; MASP: Mucin-Associated Surface Proteins; RHS: Retrotransposon Hot Spot genes.

Therefore, many efforts to unravel the structure, distribution, and functions of these multi-gene families were made. Several groups identified in the disruptive compartment of the *T. cruzi* genome multi-gene families as trans-sialidases (TSs), mucins and MASPs, whereas RHS, GP63 and DGF-1 families were located in both disruptive and core compartments [55]. Copy numbers of these multi-gene families in the genomes of strains of *T. cruzi* and B7 strain of *T. cruzi marinkellei* are displayed in Figure 3. According to data, and considering all strains as whole, the most expanded multi-gene family is the TS family, following by MASPs, RHS, mucins and DGF-1, although this is not so for all strains with available genomes. There is a high variability among strains that may be related to a strain-specific genetic profile, the accuracy of the assembled genomes, and the genomic plasticity. This produces a great diversity that could explain the different infection kinetics, virulence and/or immune responses that were detected between *T. cruzi* strains [6,7,108].

Here, we focus on the principal multi-gene families in terms of diversity, abundance and function that belong to the disruptive compartment of the *T. cruzi* genome: TSs, mucins and MASPs.

### 6.1. Trans-Sialidase (TS) Family

The membrane of parasites as *T. cruzi*, *T. brucei,* or *Trypanosoma rangeli* (*T. rangeli*) is covered by many surface proteins, and most of them are TS or TS-like proteins that are critical for the interactions with the exogenous environment. The TS family is much smaller in *T. brucei* than in *T. cruzi*, and it is absent in *Leishmania major* [42,46,109]. In *T. cruzi*, TS members are localized on the membrane surface of metacyclic, bloodstream trypomastigotes, and intracellular amastigotes and are involved in host-parasite interaction processes [110,111,112,113]. They can present a glycosylphosphatidylinositol (GPI) anchor, although this can be removed by the action of a phosphatidylinositol phospholipase C, and then TSs can be released into the bloodstream. TSs are mainly distributed along the flagellum, cell body, and flagellar pocket of the parasite as mucins [114,115].

It is the largest family in *T. cruzi* considering all strains and all their members share the VTVxNVxLYNR motif [116], although some of them present a degeneration of this motif. The first estimates based on the CL Brener genome displayed that the TS family had around 1430 members and 639 pseudogenes [42,112,117] and subsequent studies with strains as Y, Dm28c, TCC or Sylvio X10/1 obtained similar numbers. However, Bug2148 strain displayed 2325 copies [47], almost the double, which could be caused by the hybrid origin of the strain, although the percentage of TS genes with respect to the total of the genetic content is very similar to other strains. Moreover, many TSs are found near the telomeric and sub-telomeric regions, which may cause collapsed assemblies and lead to under or over-representations of the genes. This implies that part of the TS expansion is due to their chromosomal location as we explained before. The other reason is the host immune system pressure to which the TSs are exposed [75] since they are targets of both humoral and cell-mediated immune responses [112].

The best-characterized function of this family is the trans-sialidase catalytic activity, which was first described in 1980 [118]. Posterior studies demonstrated that *T. cruzi* is unable to synthesize their own sialic acids and uses the TSs to incorporate sialic acids from host-cell sialoglycoconjugates to acceptor molecules of their membranes as mucins [119,120,121,122,123,124]. This sialylation confers a negatively charged coat that protects the trypomastigotes from being killed by human anti-α galactosyl antibodies [125]. A neuraminidase was described in TSs, although it is only active when suitable Gal acceptors are present. It was suggested that this neuraminidase activity just represents around 5% of the total activity of the TS enzyme [126].

Other studies suggest that the TS activity has a key role during the *T. cruzi* infection for parasite survival and the establishment of an effective infection [127]. TSs can interact with different cells from the mammalian hosts, as thymocytes, CD4^+^ and CD8^+^ T cells, B cells, cardiac fibroblasts, endothelial cells, platelets, neurons, and Schwann cells [117]. However, the critical residues that are necessary for the catalytic activity were identified just in a few genes and other roles related to host-ligand interactions and immune regulation were proposed [110,116]. Therefore, renaming this protein family would be advisable since not all their members have TS activity.

**Figure 4:**
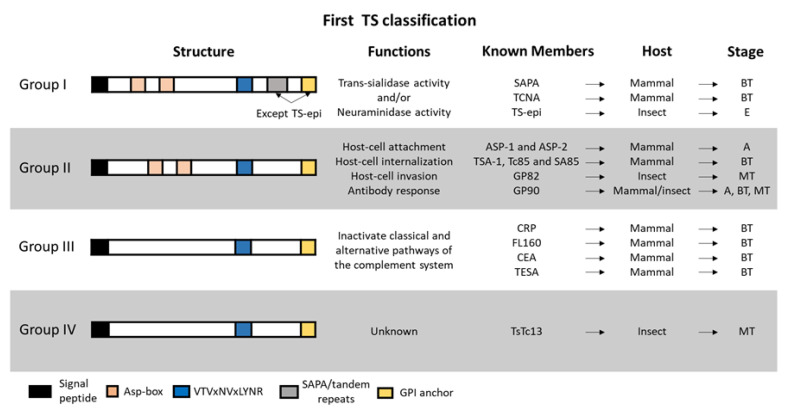
First classification of TS members. Four groups were described according to their sequence similarity and functional properties. The structure and functions of each group are displayed as well as the known members with their host and parasite-stage in which they are expressed. BT: bloodstream trypomastigotes; A: amastigotes; MT: metacyclic trypomastigotes; E: epimastigotes.

Genes encoding TS or TS-like genes were first classified into four groups according to their sequence similarity and functional properties [112,128,129] (Figure 4). TSs of the group I have trans-sialidase and/or neuraminidase catalytic activities [130] and were described in *T. rangeli* too [131]. Interestingly, *T. rangeli* lacks the trans-sialidase activity, retaining only the sialidase [132]. Some of the group I members in *T. cruzi* were the SAPA (shed acute-phase antigen), TS-epi, and TCNA (*T. cruzi* neuraminidase) proteins [116,129], which have active trans-sialidase and neuraminidase activities and are expressed in trypomastigotes [133] (except TS-epi, which is expressed and active in epimastigotes). Both SAPA and TCNA have an N-terminal catalytic region and a C-terminal extension with a tandem repeat of 12 amino acids (SAPA repeats), which consensus sequence is DSSAH [S/G]TPSTP [A/V], and a GPI anchor [134]. Conversely, TS-epi lacks the SAPA repeats and the GPI anchor [135].

TSs of group II are expressed in trypomastigotes and intracellular amastigotes and were also described in *T. rangeli* [136]. This group comprises members of the so-called GP85 glycoproteins (ASP-1, ASP-2, TSA-1, Tc85, SA85, GP82, and GP90 among others) [137] which are related to host-cell attachment [138,139,140,141,142], strong antibody responses in mice and humans [143,144,145] and *T. cruzi* internalization and invasion [141,146,147,148]. They shared with the TSs of group I, apart from the common TS motif VTVxNVxLYNR, the motifs known Asp-box (SxDxGxTW) and the C-terminal GPI anchor.

Group III is formed by TSs found in bloodstream trypomastigotes as CRP, FL160, CEA, and Trypomastigote Excreted-Secreted Antigens (TESA), which can inactivate both the classical and the alternative pathways of the complement system protecting the parasite from lysis [149,150,151]. In addition, the TS group IV have TSs with the characteristic motif VTVxNVxLYNR, but with unknown functions.

**Figure 5:**
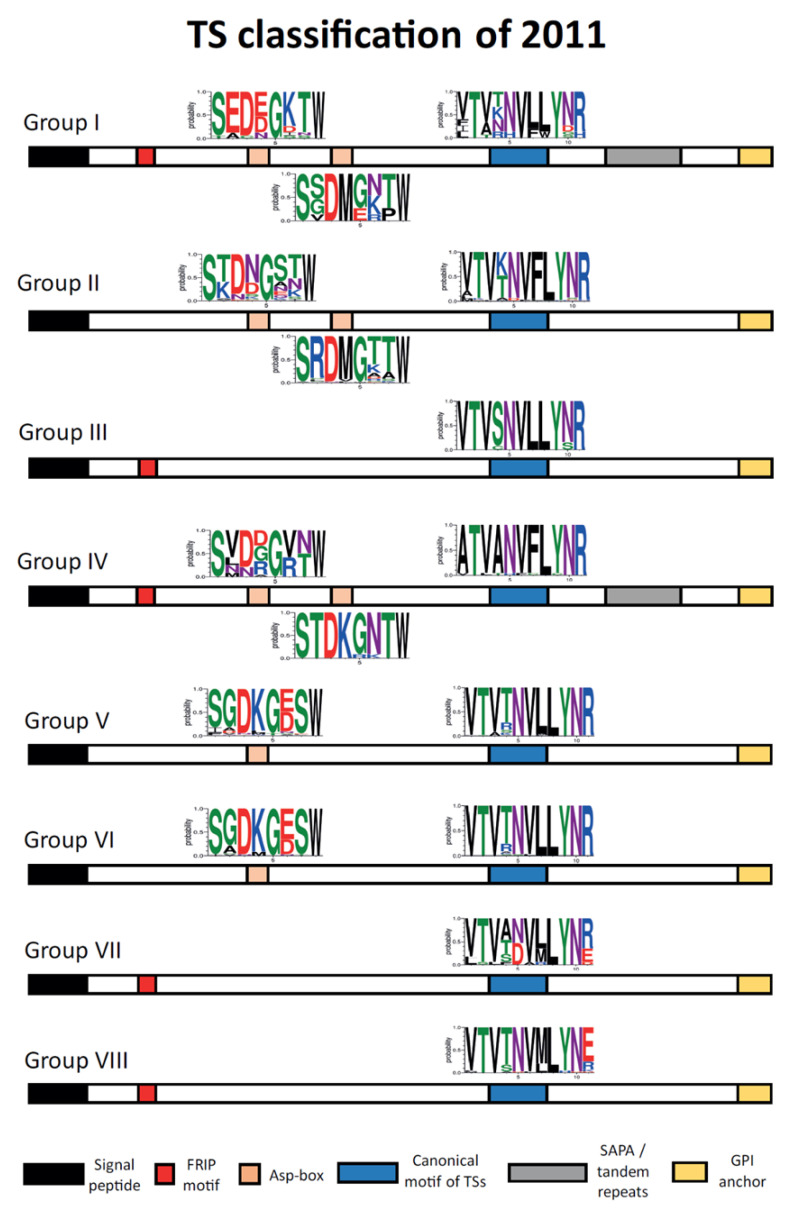
Classification of 2011 of TS members according to a sequence cluster analysis. Each group is defined by specific motifs. Logos of each Asp-box and canonical TS motifs are displayed. Adapted from Freitas, L. M. et al., 2011 [110].

However, a study of 2011 with the CL Brener strain established a different classification in eight groups by a sequence cluster analysis [110]. The sequence structure of this classification is displayed in Figure 5. The TSs of each group are defined by specific motifs and show specific activities, being the groups II and V those with more members (around 70% of the TSs in the study). Nevertheless, in databases many members of the TS family are annotated in the *T. cruzi* genomes only as trans-sialidase, without the group they belong to, making more difficult to work with this type of complex sequences.

Interestingly, phylogenetic analysis with several species of the *T. cruzi* clade and *T. brucei* showed that the variability of the TS-like sequences seems to be consistent with the aggrupation into eight groups. The detection of each TS group in each specie is displayed in Table 2. Group I TSs were found in all the species, in which two clades were established: the *T. brucei* clade with TSs of *T. brucei*, *T. congolense,* and *T. vivax* among others, and the *T. cruzi* clade with TSs of *T. cruzi*, *T. c. marinkellei* and *T. conorhini* among others [107]. It is important to remember that the TSs of group I are active catalytically, therefore it might be possible that other species just require this type of enzymatic function for their viability, while *T. cruzi* needs more TSs with other functions as interaction with host-cell ligands or immune evasion. Furthermore, other studies revealed that sialidases/sialidase-like proteins similar to all *T. cruzi* TS groups exist in *T. rangeli*, although this parasite exhibits fewer members of the trans-sialidase/sialidase family than *T. cruzi* [152,153]. TSs of group II that belong to different DTU strains were also analyzed in a new phylogenetic tree [113]. The results clustered together strains of the same DTU suggesting that TS group II genes might be used as markers for *T. cruzi* genotyping.

In this classification of eight groups, SAPA, TCNA, and TS-epi that are active TSs belonging to the previously defined group I, clustered together in the new group I, in which not all the members displayed the catalytic sites. ASP-2, Tc85, SA85, GP82, and GP90, which belonged to the previously defined group II and are related to host-cell attachment and invasion, were also classified in the new group II. In addition, finally, FL160 and other TSs involved in the complement system inhibition of the previous group III, clustered in the new group III too.

Regarding the common motifs in these TS groups, all of them have the canonical TS motif (VTVxNVxLYNR), although some variations exist (Figure 5). In fact, there is a small motif (FLY) inside the sequence that can act as a virulence factor [154], and it is only present in group II above all and group IV suggesting a host-cell attachment role in these groups. The Asp-box, previously described in viral and bacterial sialidases as SxDxGxTW [155], appears in some TSs of groups I, II, IV, V, and VI with some variations from the consensus sequence. Most of these TSs have one or two Asp-box, but a few displayed three. The function of this motif in *T. cruzi* remains unknown although it was hypothesized that TSs with these Asp-box could be more capable of binding carbohydrate molecules. The FRIP motif (with the pattern xRxP), which is located upstream the Asp-boxes and involved in binding the carboxylate group of sialic acid [156], was found in groups I, III, IV, VII, and VIII. This implies that although some TSs of these groups are enzymatically inactive, they still preserve carbohydrate-binding properties that could be important for the interaction with the host-cell [157,158]. Finally, tandem repeats, as SAPA repeats, were only found in groups I and IV. Interestingly, this classification forms three different patterns of motif occurrence. Groups I and IV have the most complex structure with all the previously described motifs, despite a few variations in the tandem repeats and the VTVxNVxLYNR motif. Groups II, V, and VI have only the Asp-box and the VTVxNVxLYNR motifs, and groups III, VII, and VIII contain only the FRIP and the VTVxNVxLYNR motifs.

**Figure 6:**
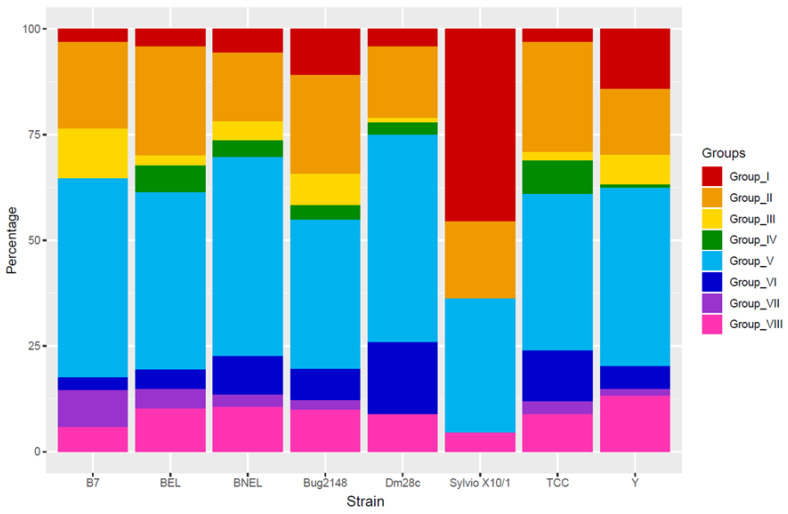
TS group distribution in genomes of different strains of *T. cruzi* and B7 strain of *T. cruzi marinkellei*. The percentage of each TS group is displayed. BNEL: CL Brener Non-Esmeraldo-like; BEL: CL Brener Esmeraldo-like.

A recent study evaluated the presence of each group in different *T. cruzi* strain genomes [47]. Considering these results and the new genomes of Dm28c and TCC (Figure 6), the TS group V is the most expanded, with the only exception of Sylvio X10/1 strain. TS group V was associated with antigenic variation allowing the adaptation of the parasites to the host environment [110]. TS group II is the second most abundant cluster, which contains trans-sialidases with host-interaction functions, and TS group I is the most expanded just in Sylvio X10/1 strain. Interestingly, TS group I that have the enzymatically active trans-sialidases, is much less abundant as predicted among strains. TS group III contains trans-sialidases that inhibit the complement pathways, and the different percentage of these trans-sialidases between strains could explain their different sensibility to the complement lysis. Finally, TS groups IV and VII are the less expanded, being absent in some strains as Sylvio X10/1, B7, or Dm28c. Therefore, this fact, in addition to the distinct distribution of Sylvio X10/1, could be caused by the quality of the assembled genomes and/or the annotation of them, being impossible to discard the presence of those TS groups or a few differences in the percentage of each TS group among strains.

### 6.2. Mucins

This is the most expressed family in the *T. cruzi* membrane and the fourth largest gene family, although 25% of them are non-functional pseudogenes [47,159]. Mucins that bear a dense array of oligosaccharides *O*-linked to serine and/or threonine residues, have two main functions: to protect the parasite from the defensive mechanisms of the host and to ensure the attachment and invasion of specific host cells [160]. These proteins are the principal acceptors of sialic acid in the parasite membrane [161] and they were classified in two subfamilies (TcMUC and TcSMUG) according to structural and biological criteria [162,163]. TcMUC proteins are only expressed in the mammalian stages of the parasite and TcSMUG in the insect-dwelling forms [160,164,165]. TcMUC proteins displayed more diversity than TcSMUG proteins and this is associated with their chromosome localization near to the telomeric regions and the immune system pressure that they suffer in the mammalian hosts [47,165].

TcSMUG (*T. cruzi* small mucin-like genes) subfamily is composed of two groups of genes, named L (large) and S (small), with differences in the genomic structure [166]. Considering the coding region, sequences of TcSMUG S and TcSMUG L display > 80% identity. TcSMUG S genes were identified as the backbone for the GP35/50 mucins that are expressed in the insect-dwelling stages [167]. GP35/50 mucins in metacyclic trypomastigotes bind to target cells to induce a bidirectional Ca^2+^ response which can contribute to the cell invasion [146]. However, the role of GP35/50 mucins in the epimastigotes is associated with protection against proteases of the insect intestinal tract [168]. Interestingly, TcSMUG S members, unlike TcSMUG L ones, are acceptors of the sialic acid residues that the TSs transferred to the parasite membrane. Otherwise, TcSMUG L products might be involved in the attachment to the luminal midgut surface of the vector and are exclusive of the epimastigote form [165,169]. Finally, some researchers saw that the expression of TcSMUG genes is post-transcriptionally regulated by AU-rich motifs of the 3′ UTR that recruit proteins to modulate the stability and translation efficiency of the mRNAs [166,170].

TcMUCs are subclassified in TcMUC I, II, and III genes. Interestingly, mucins from bloodstream trypomastigotes are called tGPI mucins and they suffer sialylation of their *O*-linked oligosaccharides by the TSs. These tGPI mucins are highly heterogeneous due to the simultaneous expression of several TcMUC I and II genes that display differences in their length, sequence, and structure of the attached oligosaccharides [162]. TcMUC II genes are quantitatively predominant over TcMUC I genes for the tGPI mucins [164].

TcMUC I members are more abundant in the amastigotes, whereas TcMUC II members are predominant in membrane lipid rafts of the bloodstream trypomastigotes [115]. TcMUC proteins contain a signal peptide, GPI anchor, and a principal central region. This central region has binding sites for N-acetylglucosamine residues and is rich in threonines. These residues are targets for the *O*-glycosylation and subsequent binding of sialic acid, which may explain why the mucins of mammalian host stages (amastigotes and bloodstream trypomastigotes) show higher glycosylation than those expressed by the epimastigotes [160]. A proportion of the TcMUC II genes are linked in the polycistronic transcription to TS genes [163,171].

In this central region TcMUC I genes have a short hypervariable (HV) section and many tandem repeats of the canonical Thr_8_-Lys-Pro_2_ sequence, although some degenerations in this sequence were described. Otherwise, TcMUC II genes have a central region with a long HV section and a few tandem repeats that are still rich in Thr and Pro. Some studies suggest that the TcMUC II genes have evolved from TcMUC I genes or vice versa. The common ancestor could be either a TcMUC I gene, which suffered a progressive expansion and diversification of its HV section or a TcMUC II gene which experienced an amplification of their original tandem repeats [163].

There is another type of mucin-like protein named TSSA (Trypomastigote Small Surface Antigen) that belongs to the TcMUC family and is present in the bloodstream trypomastigote membranes. TSSA is encoded by a single-copy gene and seems to have a role in the invasion of the host-cell as an adhesion molecule [172]. Also, it was one of the first immunological markers to allow discrimination between lineages. TSSA forms the called TcMUC group III and, unlike TcMUC I and II genes, it apparently does not display a Thr rich region [173]. Sequence analysis showed a high content of Ser and Thr residues and several signals for *O*-glycosylation [174]. However, another study described TSSA as a hypoglycosylated molecule [175], therefore further research is needed to elucidate its glycan composition and structure.

### 6.3. MASPs

MASPs (Mucin-Associated Surface Proteins) have a structural similarity to TcMUC II proteins and their expression seems to be up-regulated in mammal-dwelling stages [71]. They are the second largest gene family in the *T. cruzi* genome and received that name from their cluster position among large TS and mucin gene groups. MASPs are characterized by highly conserved N- and C- terminal domains, a GPI anchor, and a variable and repetitive central region [176].

According to some studies the MASP family constitutes about 6% of the parasite haploid genome and comprises between 500 and more than 1000 members varying among strains [47,55]. As in the TS family, the hybrid strain Bug2148 displays around the double of genes that Dm28c, Sylvio X10/1, or Y strains. The high variability of this family is not only due to the telomeric location of some of their members, and some researchers suggested that other mechanisms may exist. The high conservation of some motifs of the UTR sequences of these genes could contribute as sites for homologous recombination. It was suggested that one of the main mechanisms could be the retrotransposition by mobile elements of the TcTREZO type, specific of this gene family with its insertion sites at the conserved 5′ and 3′ ends [177].

MASPs have sites for both *N*- and *O*-glycosylation which undergo extensive post-translational modifications and were detected in trypomastigotes, amastigotes, and epimastigotes [178]. However, they seem to be overexpressed in the infectious stages (metacyclic and bloodstream trypomastigotes) and a critical role in the invasion process favoring the endocytosis was suggested. Other researchers have speculated that changes in the repertoire of MASP antigenic peptides could contribute to the evasion of the host immune system during the acute phase of Chagas disease [179]. Otherwise, antibodies against a specific MASP member can produce a decrease in the parasite internalization. MASP overexpression in the amastigote membrane before the binary fission suggests that some of these proteins play a major biological role in the survival and multiplication of the intracellular amastigotes [180].

## 7. Conclusions

Genomic studies are essential for the understanding of the *T. cruzi* pathogenicity and biology. The new sequencing technologies have contributed to improve the quality of several genomes of different strains and to elucidate the broad genetic diversity and complexity of this parasite. In this regard, the combination of long- and short-read sequencing methods may overcome the problems in the genome assembly and annotation due to the high intrinsic genome complexity of *T. cruzi*. Therefore, we need to wonder if the CL Brener genome is the best as reference in the databases, considering the new genomes of other strains that were obtained with these sequencing methods that have improved both assembly and annotation processes.

The study of the different repetitive sequences, recombination processes, and gene expansion events of the *T. cruzi* genome shows that genome plasticity plays a key role as a survival strategy during the life cycle of the parasite. Therefore, further research is needed to understand these relevant processes of the parasite biology.

Regarding the principal multi-gene families of *T. cruzi*, this parasite presents a wide variety of surface proteins with important roles in its life cycle. Due to the fact of genome plasticity, these multi-gene families have suffered an expansion and constant evolution that have increased the *T. cruzi* ability of adaptation, survival, and infection of both insect and mammalian hosts.

Altogether, the recent advances in trans-sialidases, mucins, and other multi-gene families can positively increase the current knowledge of host-parasite interactions and will allow the design of effective drugs against the Chagas disease. However, due to the genome complexity of the parasite more studies to unravel the specific structure and functions of those proteins will be needed. In this regard, high-throughput technologies will be useful to establish the development and evolution of multi-gene families of *T. cruzi*.

## Figures and Tables

**Table 1:** Data of the most recent genomes of the best-studied strains of *T. cruzi* and the B7 strain of *T. cruzi marinkellei*. BNEL: CL Brener Non-Esmeraldo-like; BEL: CL Brener Esmeraldo-like; PacBio: Pacific Biosciences. Contig N50: is a statistic median such that the 50% of the whole assembly is contained in contigs equal to or larger than this value.

Strain	DTU	Size (Mbp)	Contigs	Contig N50	%GC	Date of Version	Sequencing Method	References
G	I	25.17	1450	74,655	47.40	November 2018	Roche 454	[50]
Dm28c	I	53.27	636	317,638	51.60	May 2018	Illumina + PacBio	[55]
Sylvio X10/1	I	38.59	27,019	2307	51.20	October 2012	Roche 454 + Illumina	[49,57]
Berenice	II	40.80	934	148,957	51.20	June 2020	Illumina + Nanopore	[54]
Y	II	39.34	10,127	11,782	51.43	October 2017	Illumina	[47]
231	III	35.36	8469	14,202	48.60	January 2018	Illumina	[48]
Bug2148	V	55.22	934	196,760	51.63	October 2017	PacBio	[53]
CL	VI	65.00	7764	73,547	39.80	November 2018	Roche 454	[50]
TCC	VI	87.06	1236	264,196	51.70	May 2018	Illumina + PacBio	[55]
CL Brener	VI	89.94	32,746	14,669	51.70	July 2005	Sanger	[42]
BNEL	VI	32.53	41	870,934	43.94	December 2015	Sanger	[58]
BEL	VI	32.53	41	870,934	40.35	December 2015	Sanger	[58]
*T. c. marinkellei* B7 strain	---	38.65	23,154	2846	50.90	October 2012	Roche 454 + Illumina	[51]

**Table 2:** Presence of at least one member of each TS group in different *Trypanosoma* species.

		TS Groups of *T. cruzi*							
		Group I	Group II	Group III	Group IV	Group V	Group VI	Group VII	Group VIII
***Trypanosoma* species with TS sequence similarity**	*T. c. marinkellei*	✓	✓	✓	✓	✓	✓	✓	✓
	*T. rangeli*	✓	✓	✓	✓	✓	✓	✓	✓
	*T. conorhini*	✓	✓			✓			
	*T. dionisii*	✓	✓						
	*T. evansi*	✓							
	*T. congolense*	✓							
	*T. vivax*	✓							
	*T. grayi*	✓							
	*T. carassii*	✓							
	*T. brucei*	✓							
